# Associations between MRI T1 mapping, liver stiffness, quantitative MRCP, and laboratory biomarkers in children and young adults with autoimmune liver disease

**DOI:** 10.1007/s00261-021-03378-0

**Published:** 2021-12-21

**Authors:** Neeraja Mahalingam, Andrew T. Trout, Deep B. Gandhi, Rashmi D. Sahay, Ruchi Singh, Alexander G. Miethke, Jonathan R. Dillman

**Affiliations:** 1 Department of Radiology, Imaging Research Center, Cincinnati Children’s Hospital Medical Center, 250 Albert Sabin Way, Cincinnati, OH USA; 2 Department of Radiology, Cincinnati Children’s Hospital Medical Center, Cincinnati, OH USA; 3 Department of Radiology, University of Cincinnati College of Medicine, Cincinnati, OH USA; 4 Department of Pediatrics, University of Cincinnati College of Medicine, Cincinnati, OH USA; 5 Division of Biostatistics and Epidemiology, Cincinnati Children’s Hospital Medical Center, Cincinnati, OH USA; 6 Center for Autoimmune Liver Disease (CALD), Cincinnati Children’s Hospital Medical Center, Cincinnati, OH USA; 7 Division of Gastroenterology, Hepatology, and Nutrition, Cincinnati Children’s Hospital Medical Center, Cincinnati, OH USA

**Keywords:** Autoimmune liver disease, Children, Iron-corrected T1, Magnetic resonance cholangiopancreatography, Magnetic resonance elastography

## Abstract

**Purpose:**

Define relationships between quantitative magnetic resonance imaging (MRI) metrics and clinical/laboratory data in a pediatric and young adult cohort with autoimmune liver disease (AILD).

**Materials and methods:**

This prospective, cross-sectional study was institutional review board-approved. Patients enrolled in an institutional AILD registry were divided into groups: (1) autoimmune hepatitis (AIH) or (2) primary sclerosing cholangitis (PSC)/autoimmune sclerosing cholangitis (ASC). Participants underwent serum liver biochemistry testing and research MRI examinations, including 3D magnetic resonance cholangiopancreatography (MRCP), magnetic resonance elastography (MRE), and iron-corrected T1 mapping (cT1). MRCP + and LiverMultiScan (Perspectum Ltd., Oxford, UK) were used to post-process 3D MRCP and cT1 data. Multiple linear regression models were used to assess relationships.

**Results:**

58 patients, 35 male, median age 16 years were included; 30 in the AIH group, 28 in the PSC/ASC group. After statistical adjustments for patient age, sex, presence of inflammatory bowel disease (IBD), specific diagnosis (PSC/ASC vs. AIH), and time from diagnosis to MRI examination, left hepatic bile duct maximum diameter was a statistically significant predictor of whole liver mean cT1, cT1 interquartile range (IQR), and MRE liver stiffness (*p* = 0.01–0.04). Seven laboratory values were significant predictors of whole liver cT1 IQR (*p* < 0.0001–0.04). Eight laboratory values and right hepatic bile duct median and maximum diameter were significant predictors of liver stiffness (*p* < 0.0001–0.03).

**Conclusions:**

Bile duct diameters and multiple laboratory biomarkers of liver disease are independent predictors of liver stiffness and cT1 IQR in pediatric patients with AILD.

**Supplementary Information:**

The online version contains supplementary material available at 10.1007/s00261-021-03378-0.

## Introduction

Autoimmune liver diseases (AILD) include three, sometimes overlapping, conditions: autoimmune hepatitis (AIH), primary sclerosing cholangitis (PSC), and autoimmune sclerosing cholangitis (ASC) [1–3]. These diseases can have a significant impact on liver function and health, often causing progressive fibrosis and, in some cases, cirrhosis and end-stage liver disease [3, 4]. More accurate diagnosis and monitoring could potentially allow more precise patient management and improve outcomes [1, 4, 5].

There is increasing evidence that quantitative MRI techniques, such as MR elastography (MRE) and T1 mapping, can be used to non-invasively measure the severity of chronic liver disease and predict liver histologic fibrosis stage [6–8]. Liver stiffness measured from MRE has been shown to correlate with biliary stricture severity in adult patients with PSC [8, 9]. Additionally, MRE-derived liver stiffness has been shown to positively correlate with advanced stages of fibrosis [10, 11]. T1 relaxation times have also been shown to correlate well with histologic markers of pathology and to increase with advanced liver fibrosis and inflammation [7, 12–14]. These studies are mainly in adult populations, with only limited data available in pediatric populations [2, 15, 16].

Magnetic resonance cholangiopancreatography (MRCP) has been shown to sufficiently diagnose PSC in children, with sensitivities of 81–84% and specificity up to 100% [17, 18]. Recent evidence has also shown that quantitative metrics obtained from conventional anatomic 3D MRCP images of the biliary tree can be used to assess patients with AILD. Gilligan et al. [19] showed that quantitative analysis of 3D MRCP data can discriminate between pediatric patients with AIH and PSC/ASC and that quantitative MRCP metrics (e.g., number and total length of bile duct strictures/dilations) may serve as non-invasive biomarkers of AILD.

The objective of this study was to investigate relationships between quantitative MRI metrics of liver disease including (1) MRE-derived liver stiffness, (2) T1 relaxation measurements, and (3) quantitative 3D MRCP measurements, and clinical and laboratory data in a pediatric and young adult AILD cohort.

## Materials and methods

This single-center, prospective, cross-sectional, Health Insurance Portability and Accountability Act (HIPAA)-compliant pilot study was approved by the institutional review board at Cincinnati Children's Hospital Medical Center (CCHMC).

Patients up to 25 years of age with AILD (including AIH, PSC, and ASC) that were enrolled into an institutional AILD registry were included in this study. A group of pediatric hepatologists at CCHMC assigned the registry participants with a diagnosis of AIH, PSC, or ASC according to clinical guidelines [3, 19–21]. Written informed consent, and assent as appropriate, were obtained. Patients with any other form of liver disease were excluded from the registry.

### MRI acquisition protocol

All registry participants underwent research MRI examinations of the liver at the time of registry enrollment. Subsequent research MRI examinations using the same protocol and scanner were performed 12 and 24 months later. The current study includes baseline MRI examinations acquired on a 1.5 T scanner (Ingenia; Philips Healthcare, Best, The Netherlands) using a 16-channel phased-array anterior (torso) surface coil as well as scanner table built-in spine coils (12-channels). The imaging protocol and parameters for the broader registry study have been previously published [2, 19]. Pertinent quantitative sequences for the current study include the following:

1. Three-dimensional (3D) fast spin-echo (FSE) MRCP using respiratory triggering; the belt used to detect breathing was placed over the upper abdomen.
2. Iron (T2*)-corrected T1 mapping (cT1) of the liver using a modified Look Locker (MOLLI) pulse sequence approach and complex chemical shift MRI [6].
3. Two-dimensional (2D) gradient recalled echo (GRE) MRE of the liver (active driver frequency = 60 Hz), with the passive driver placed over the right upper quadrant of the abdomen and secured in place with a Velcro strap.

### Clinical and laboratory data collection

Pertinent demographic and clinical data were recorded from the AILD registry. The following clinical data were recorded: patient age, sex, clinical diagnosis (AIH, PSC, or ASC), time between diagnosis and research MRI (in months), and presence of inflammatory bowel disease (IBD). The following laboratory measurements also were recorded from the time of the research MRI: total bilirubin, alanine aminotransferase (ALT), aspartate aminotransferase (AST), gamma-glutamyl transferase (GGT), platelets, fibrosis-4 (FIB4) score, AST to platelet ratio index (APRI), alkaline phosphatase (ALP), and matrix metalloproteinase 7 (MMP7), a novel marker of bile duct injury and liver fibrosis in AILD [22]. Methods for calculating FIB4 score and APRI have been previously published [23].

### MR image post-processing

Perspectum Ltd. (Oxford, United Kingdom) provided image post-processing tools and analyses for quantitative 3D MRCP metrics and iron-corrected T1 (cT1) relaxation measurements at no cost through a formal research agreement. Their analysts were blinded to all patient information, including clinical diagnosis as well as laboratory, clinical, and other imaging data.

#### 3D MRCP

3D MRCP images were processed by a single operator at Perspectum using the MRCP + tool. This operator has more than 5 years of experience using MRCP + . To achieve this, bile ducts were identified through a series of signal enhancement and thresholding steps to detect tubular structures and generate a 3D model of the biliary tree [19]. The following metrics were then quantified: biliary tree volume; maximum and median diameters of the common bile duct, left hepatic bile duct, and right hepatic bile duct; total number of ducts in the 3D biliary tree model, defined as the total number of branches in the modeled tree; total length of ducts; total number of strictures, defined as local minima that were more than 30% narrower than neighboring maxima, total length of duct strictures; total number of dilations, defined as local maxima that were more than 30% wider than neighboring minima; and total length of duct dilations [19]. Diameters and lengths were reported in millimeters (mm).

#### cT1

Region-of-interest (ROI) and whole liver cT1 values were provided by Perspectum using LiverMultiScan [6, 24, 25], with values derived by a single observer with more than 5 years of experience using LiverMultiScan. For ROI measurements, three equally sized circular ROIs, with a diameter of 15 mm, were drawn in the right hepatic lobe of the liver on one representative axial slice, while avoiding major blood vessels and bile ducts. Voxel-by-voxel cT1 values were also calculated for the whole liver on four representative axial slices. The mean cT1 for the three circular ROIs and the mean, median, and interquartile range (IQR) of whole liver cT1 measurements across the four axial slices were calculated.

#### 2D GRE MRE

MREplus + prototype software (Resoundant Inc., Rochester, Minnesota) was utilized to measure liver stiffness values from MRE data. Under the supervision of an image analyst in the Department of Radiology who was blinded to clinical data and other imaging data, ROIs were automatically placed on elastograms with 90% confidence threshold masks across four representative axial slices. The weighted mean of the mean liver shear stiffness values (in kPa) across the four axial slices was calculated for each patient.

**Fig. 1:**
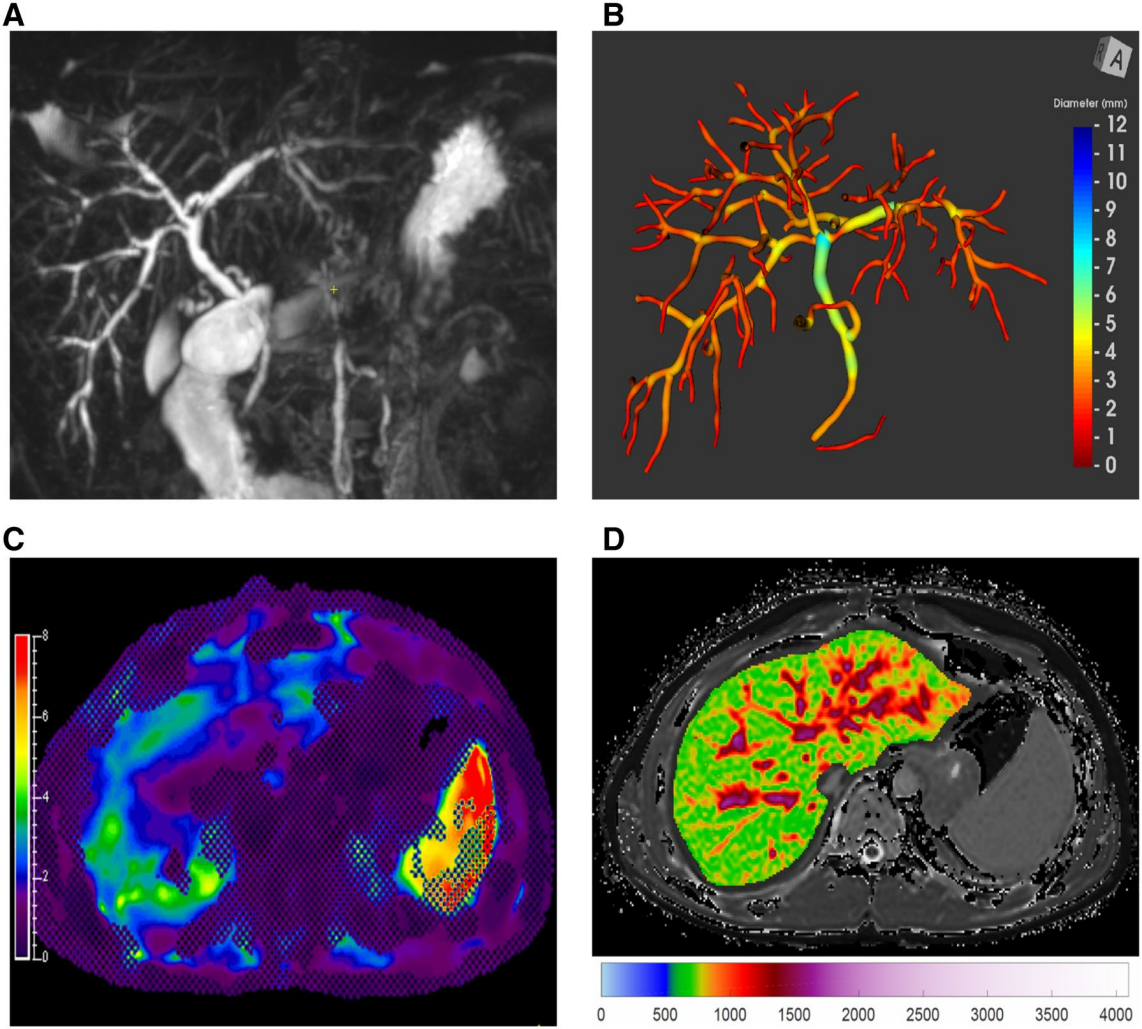
13-year-old male with autoimmune sclerosing cholangitis. **a** Maximum intensity projection 3D MRCP image. **b** Corresponding 3D biliary tree model extracted from 3D MRCP image using MRCP + . **c** MR elastogram of the liver (units of kPa). **d** Iron-corrected T1 (cT1) map of the liver (units of ms)

Representative images of the quantitative MRI sequences assessed in this study are provided in Fig. 1.

### Statistical analysis

The patient cohort was divided into two groups, (1) AIH and (2) PSC/ASC. Patients with PSC/ASC were grouped into one cohort due to similarities in MRCP findings and clinical outcomes [3, 19]. Continuous variables were summarized as median, first quartile, and third quartile values. Variables that were not normally distributed were log transformed using base10 for analyses (Supplementary Table 1). Group differences in age, MRE liver stiffness, cT1, quantitative MRCP metrics, and laboratory values between the AIH and PSC/ASC groups were examined using the Mann–Whitney *U* test. Categorical variables were presented as frequency counts and percentages; group differences were determined using Chi-square/Fisher’s exact test, as applicable.

Five variables were considered primary outcome variables: liver stiffness, ROI-based mean cT1, whole liver mean cT1, whole liver median cT1, and whole liver cT1 IQR. The following variables were considered potential predictor variables: demographic data, quantitative MRCP metrics, and serum laboratory values. Univariate associations between outcomes and predictor variables for the entire study cohort and for the AIH and PSC/ASC groups individually were assessed using Pearson correlation coefficients (*r*). Relationships between three individual outcome variables (liver stiffness, whole liver mean cT1, and whole liver cT1 IQR) and potential predictor variables were further examined using multiple linear regression models, adjusting for patient age, sex, the presence of IBD, specific diagnosis (AIH vs. PSC/ASC), and time from diagnosis to research MRI examination. Stratified multiple linear regression models by diagnosis (AIH vs PSC/ASC) were also run, while also adjusting for patient age, sex, presence of IBD, and time from diagnosis to research MRI examination.

*p* values were not adjusted for multiple comparisons as this was an exploratory assessment. *p* less than 0.05 was considered statistically significant. All statistical testing was carried out on SAS version 9.4 (SAS Institute, Cary, North Carolina).

## Results

**Fig. 2:**
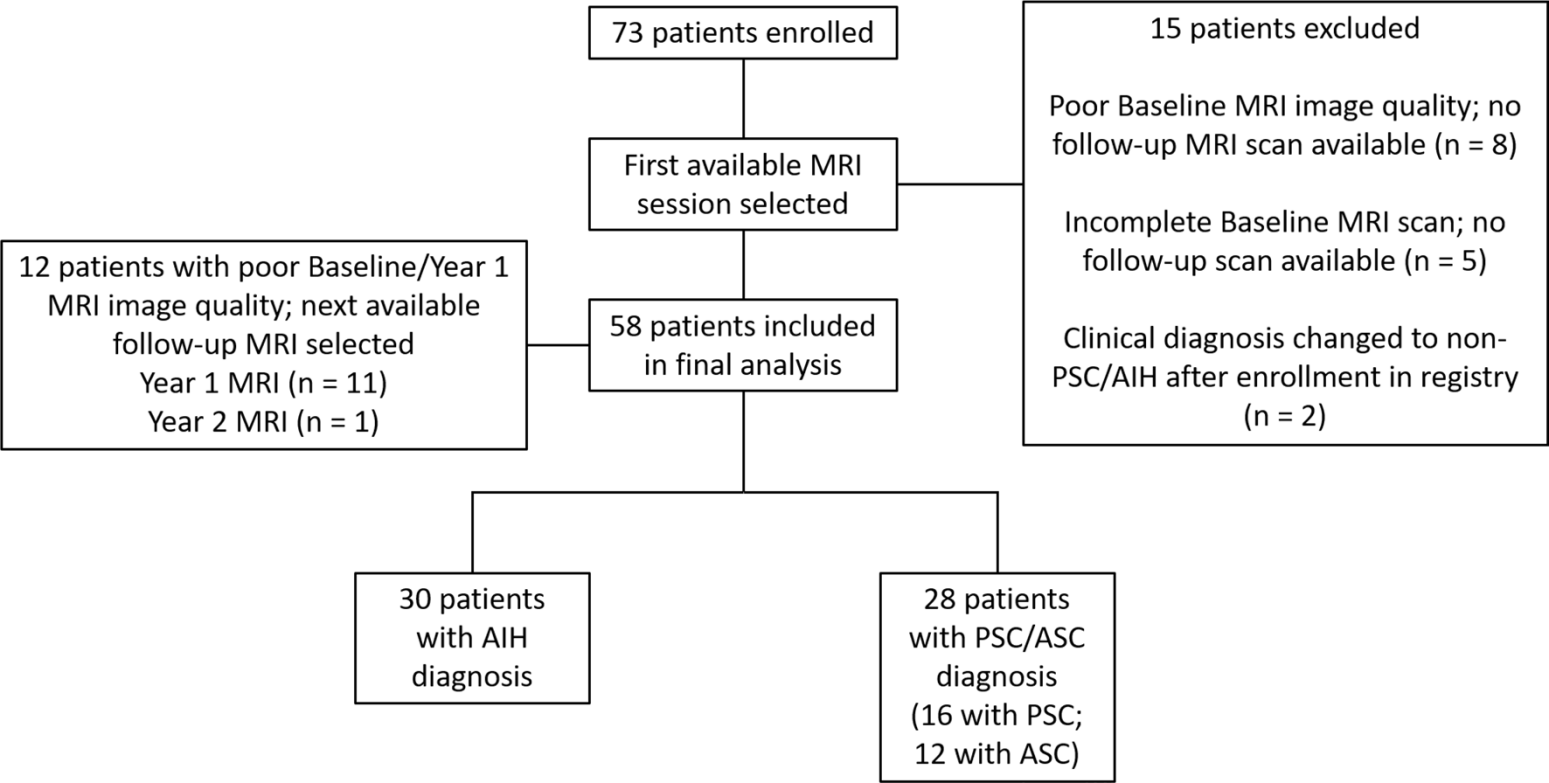
Patient/MRI examination selection flow diagram. *MRI* magnetic resonance imaging, *AIH* autoimmune hepatitis, *ASC* autoimmune sclerosing cholangitis, *PSC* primary sclerosing cholangitis

Seventy-three patients were eligible for inclusion in the current study. Fifteen patients were excluded for reasons detailed in Fig. 2. Of the remaining 58 patients, 12 had poor quality baseline MRI examinations (i.e., did not allow quantitative 3D MRCP processing due to artifacts), which were replaced with the next available research MRI examination at follow-up year 1 or year 2. Substitution of examinations was deemed acceptable because this is a cross-sectional study and includes patients at varying timepoints in their disease course at registry enrollment, ranging from newly diagnosed to end-stage liver disease.

Detailed patient demographic information and laboratory value summary statistics are given in Table 1. The median age of the AIH group (*n* = 30) was 16.0 years, and the median age of the PSC/ASC group (*n* = 28) was 15.5 years; cohort age was not statistically significantly different (*p* = 0.56). There were no differences in laboratory values between cohorts, with the exception of platelet count (*p* = 0.01) and MMP7 levels (*p* = 0.04).

**Table 1:** Demographic and laboratory value summary statistics for the study cohort

Variable	Diagnostic groups		
	AIH (*N* = 30)	PSC/ASC (*N* = 28)	*p* value
	Median (Q1, Q3)	Median (Q1, Q3)	
Age (years)	16 (13, 18)	15.5 (13, 17.5)	0.56
Number of males^a^	15	20	0.1*
Time from diagnosis (months)	12 (1, 47)	6.5 (2, 44.5)	0.79
Patients taking corticosteroid^a^	9	6	0.46*
Patients taking azathioprine^a^	11	3	0.02*
Patients taking ursodeoxycholic acid^a^	4	16	0.001*
Patients with IBD^a^	2	20	< 0.0001*
Total bilirubin (mg/dL)	0.55 (0.4, 0.8)	0.5 (0.3, 0.9)	0.59
ALT (units/L)	42 (23, 70)	41 (27, 80.5)	0.66
AST (units/L)	34 (25, 41)	30.5 (21, 54.5)	0.99
GGT (units/L)	35.5 (15, 76)	47 (24, 227)	0.07
Platelets (× 10^9^/L)	232.5 (172, 275)	275.5 (221.5, 341.5)	**0.01**
APRI	0.47 (0.29, 0.93)	0.36 (0.2, 0.57)	0.16
FIB4	0.31 (0.14, 0.66)	0.23 (0.14, 0.45)	0.25
ALP (units/L)	148.5 (88, 192)	206.5 (122.5, 294.5)	0.07
MMP7 (units/L)	14 (11.62, 16.49)	26.83 (13.54, 40.85)	**0.04**

Significant *p* values are indicated in bold

*IBD* inflammatory bowel disease, *ALT* alanine aminotransferase, *AST* aspartate aminotransferase, *GGT* gamma-glutamyl transferase, *APRI* AST to platelet ratio index, *FIB4* fibrosis-4 score, *ALP* alkaline phosphatase, *MMP7* matrix metalloproteinase 7

*p* values were derived using Mann–Whitney *U* test unless noted

*p value derived from Chi-square/Fisher’s exact test

^a^Number of males, patients taking corticosteroid, azathioprine, and ursodeoxycholic acid, and patients with IBD reported as counts

Summary statistics for liver stiffness, cT1 measurements, and quantitative MRCP metrics are provided in Table 2. There were no significant differences in liver stiffness or cT1 values between the AIH and PSC/ASC cohorts. All quantitative MRCP metrics, except median and maximum right hepatic bile duct diameters, were statistically significantly different between the two cohorts.

**Table 2:** MRI (liver stiffness, cT1, and quantitative MRCP) summary statistics for the overall study cohort (*n* = 58), subdivided by specific diagnosis

Variable	Diagnostic groups		
	AIH (*N* = 30)	PSC/ASC (*N* = 28)	*p* value
	Median (Q1, Q3)	Median (Q1, Q3)	
Liver stiffness (kPa)	2.53 (2.02–3.23)	2.65 (2.24–3.36)	0.38
Mean cT1 in liver ROI (ms)	815.12 (728.84–862.29)	759.69 (717.67–813.12)	0.12
Mean cT1 in whole liver (ms)	895.37 (826.48–934.98)	854.53 (818–894.52)	0.09
Median cT1 in whole liver (ms)	816.5 (744–861)	760.0 (729.5–815)	0.06
cT1 IQR in whole liver (ms)	128.5 (113–171)	142.0 (116.5–164.5)	0.68
Biliary tree volume (mL)	3.8 (2.3–6)	7.4 (3.8–12.7)	**0.002**
Common bile duct median diameter (mm)	3.6 (3–4.4)	4.55 (3.5–5.55)	**0.003**
Common bile duct maximum diameter (mm)	4.9 (4–6.4)	6.5 (5.1–8.5)	**0.002**
Left hepatic bile duct median diameter (mm)	3.6 (2.7–4)	4.1 (3.4–5)	**0.01**
Left hepatic bile duct maximum diameter (mm)	4.4 (3.8–4.8)	5.3 (4.5–6)	**0.01**
Right hepatic bile duct median diameter (mm)	3.2 (2.3–4)	2.8 (1.8–4.3)	0.82
Right hepatic bile duct maximum diameter (mm)	3.9 (2.8–4.5)	4.4 (2.4–6.2)	0.58
Total number of bile ducts (total number of branches in the modeled tree)	22 (11–40)	50 (26.5–73)	**0.002**
Total length of bile ducts (biliary tree) (mm)	444.5 (229.7–910.3)	1221.2 (546.0–1933.1)	**0.005**
Total number of duct strictures	2 (1–4)	5 (3.5–8.5)	**0.0003**
Total length of duct strictures (mm)	14.9 (5.3–34.6)	41.5 (25.8–64.4)	**0.001**
Total number of duct dilations (mm)	3 (1–6)	9 (5.5–14)	** < 0.0001**
Total length of duct dilations (mm)	19.7 (7.5–34.9)	62.7 (36.4–91.6)	** < 0.0001**

Significant *p* values are indicated in bold

*MRE* MR elastography, *cT1* iron (T2*)-corrected T1 mapping, *ROI* region of interest, *IQR* interquartile range

*p* values derived from Mann–Whitney *U* test

### Univariate correlations

Univariate relationships between outcome variables and predictor variables for the entire study cohort are presented in Table 3. Results for univariate correlation analyses for the AIH and PSC/ASC groups are provided in Supplementary Tables 2 and 3.

**Table 3:** Univariate correlations between liver stiffness and cT1 measurements and predictor variables in overall study cohort (AIH, PSC, and ASC) (*n* = 58)

Predictor variables	ROI-based mean cT1 (ms)	Whole liver mean cT1 (ms)	Whole liver median cT1 (ms)	Whole liver cT1 IQR (ms)^a^	MRE liver stiffness (kPa)^a^
Common bile duct median diameter (mm)	− 0.01 [− 0.27, 0.25] (n.s)	0.04 [− 0.22, 0.30] (n.s)	− 0.02 [− 0.27, 0.24] (n.s)	0.20 [− 0.07, 0.43] (n.s)	− 0.01 [− 0.27, 0.24] (n.s)
Common bile duct maximum diameter (mm)	0.06 [− 0.20, 0.32] (n.s)	0.11 [− 0.16, 0.35] (n.s)	0.06 [− 0.20, 0.31] (n.s)	0.21 [− 0.05, 0.45] (n.s)	0.13 [− 0.14, 0.37] (n.s)
Left hepatic bile duct median diameter (mm)	0.15 [− 0.12, 0.40] (n.s)	0.29 [0.02, 0.51] (0.04)*	0.21 [− 0.06, 0.45] (n.s)	0.32 [0.06, 0.54] (0.02)*	0.27 [0.01, 0.50] (0.04)*
Left hepatic bile duct maximum diameter (mm)	0.19 [− 0.08, 0.43] (n.s)	0.34 [0.08, 0.56] (0.01)*	0.24 [− 0.03, 0.48] (n.s)	0.38 [0.13, 0.59] (0.004)*	0.28 [0.02, 0.51] (0.04)*
Right hepatic bile duct median diameter (mm)	0.34 [0.08, 0.55] (0.01)*	0.35 [0.10, 0.56] (0.008)*	0.31 [0.06, 0.53] (0.02)*	0.23 [− 0.04, 0.46] (n.s)	0.33 [0.08, 0.54] (0.01)*
Right hepatic bile duct maximum diameter (mm)	0.33 [0.08, 0.55] (0.01)*	0.35 [0.09, 0.56] (0.008)*	0.30 [0.05, 0.52] (0.02)*	0.23 [− 0.03, 0.46] (n.s)	0.31 [0.06, 0.53] (0.02)*
Biliary tree volume (mL)^a^	− 0.05 [− 0.30, 0.21] (n.s)	0.04 [− 0.22, 0.30] (n.s)	− 0.05 [− 0.30, 0.21] (n.s)	0.12 [− 0.14, 0.37] (n.s)	0.08 [− 0.18, 0.34] (n.s)
Total number of bile ducts^a^	− 0.08 [− 0.33, 0.18] (n.s)	− 0.02 [− 0.28, 0.24] (n.s)	− 0.08 [− 0.33, 0.18] (n.s)	0.01 [− 0.25, 0.27] (n.s)	− 0.001 [− 0.26, 0.26] (n.s)
Total length of bile ducts (mm)^a^	− 0.03 [− 0.29, 0.23] (n.s)	0.03 [− 0.23, 0.29] (n.s)	− 0.04 [− 0.29, 0.22] (n.s)	0.04 [− 0.22, 0.30] (n.s)	0.01 [− 0.25, 0.27] (n.s)
Total number of duct strictures^a^	− 0.03 [− 0.29, 0.23] (n.s)	0.10 [− 0.17, 0.35] (n.s)	− 0.01 [− 0.27, 0.25] (n.s)	0.22 [− 0.04, 0.45] (n.s)	0.19 [− 0.07, 0.43] (n.s)
Total length of duct strictures (mm)^a^	− 0.01 [− 0.27, 0.25] (n.s)	0.12 [− 0.14, 0.37] (n.s)	0.02 [− 0.24, 0.28] (n.s)	0.23 [− 0.03, 0.46] (n.s)	0.16 [− 0.10, 0.40] (n.s)
Total number of duct dilations^a^	− 0.03 [− 0.28, 0.23] (n.s)	0.06 [− 0.21, 0.31] (n.s)	− 0.02 [− 0.28, 0.24] (n.s)	0.18 [− 0.08,0.42] (n.s)	0.23 [− 0.03, 0.46] (n.s)
Total length of duct dilations (mm)^a^	− 0.006 [− 0.26, 0.25] (n.s)	0.08 [− 0.18, 0.34] (n.s)	− 0.01 [− 0.27, 0.25] (n.s)	0.17 [− 0.10, 0.41] (n.s)	0.21 [− 0.05, 0.44] (n.s)
Age (years)	0.19 [− 0.07, 0.43] (n.s)	0.33 [0.08, 0.54] (0.01)*	0.20 [− 0.06, 0.43] (n.s)	0.35 [0.10, 0.56] (0.008)*	− 0.05 [− 0.30, 0.21] (n.s)
Time from diagnosis to MRI (months)	− 0.14 [− 0.39, 0.13] (n.s)	− 0.15 [− 0.40, 0.12] (n.s)	− 0.19 [− 0.43, 0.08] (n.s)	0.08 [− 0.19, 0.34] (n.s)	0.0002 [− 0.27, 0.27] (n.s)
Total bilirubin (mg/dL)^a^	0.17 [− 0.09, 0.41] (n.s)	0.32 [0.07, 0.54] (0.01)*	0.20 [− 0.06, 0.43] (n.s)	0.53 [0.32, 0.69] (< 0.0001)*	0.33 [0.08, 0.54] (0.01)*
ALT (units/L)^a^	0.21 [− 0.06, 0.44] (n.s)	0.21 [− 0.05, 0.44] (n.s)	0.19 [− 0.07, 0.43] (n.s)	0.32 [0.06, 0.53] (0.02)*	0.57 [0.36, 0.72] (< 0.0001)*
AST (units/L)^a^	0.21 [− 0.05, 0.45] (n.s)	0.26 [0, 0.48] (n.s)	0.24 [− 0.02, 0.47] (n.s)	0.39 [0.15, 0.59] (0.002)*	0.56 [0.35, 0.71] (< 0.0001)*
GGT(units/L)^a^	0.10 [− 0.17, 0.35] (n.s)	0.04 [− 0.22, 0.29] (n.s)	0.05 [− 0.21, 0.30] (n.s)	0.26 [0, 0.49] (0.04)*	0.59 [0.39, 0.74] (< 0.0001)*
Platelets ( x 10^9^/L)	− 0.09 [− 0.35, 0.17] (n.s)	− 0.23 [− 0.46, 0.03] (n.s)	− 0.15 [− 0.39, 0.11] (n.s)	− 0.50 [− 0.67, − 0.28] (< 0.0001)*	− 0.34 [− 0.55, − 0.08] (0.01)*
APRI^a^	0.27 [0.02, 0.50] (0.04)*	0.38 [0.14, 0.58] (0.003)*	0.33 [0.08, 0.54] (0.01)*	0.61 [0.42, 0.75] (< 0.0001)*	0.65 [0.47, 0.78] (< 0.0001)*
FIB4^a^	0.30 [0.05, 0.52] (0.02)*	0.44 [0.21, 0.63] (0.0005)*	0.36 [0.11, 0.56] (0.006)*	0.66 [0.49, 0.79] (< 0.0001)*	0.62 [0.43, 0.75] (< 0.0001)*
ALP (units/L)^a^	− 0.16 [− 0.40, 0.10] (n.s)	− 0.22 [− 0.45, 0.04] (n.s)	− 0.19 [− 0.42, 0.08] (n.s)	0.14 [− 0.12, 0.39] (n.s)	0.42 [0.18, 0.61] (0.001)*
MMP7 (units/L)^a^	0.15 [− 0.30, 0.54] (n.s)	0.10 [− 0.34, 0.51] (n.s)	0.16 [− 0.29, 0.56] (n.s)	0.01 [− 0.42, 0.44] (n.s)	0.51 [0.10, 0.77] (0.02)*

Pearson correlation coefficients (*r*) are presented with 95% confidence intervals in brackets and *p* values in parentheses

*cT1* iron (T2*)-corrected T1 mapping, *ROI* region of interest, *IQR* interquartile range, *MRE* MR elastography, *n.s* not significant (*p* ≥ 0.05), *ALT* alanine aminotransferase (units/L), *AST* aspartate aminotransferase (units/L), *GGT* gamma-glutamyl transferase (units/L), *APRI* AST to platelet ratio index, *FIB4* fibrosis-4 score, *ALP* alkaline phosphatase (units/L), *MMP7* matrix metalloproteinase 7 (units/L)

*Statistically significant (bold)

^a^Variables examined as log10 value

### Associations between outcome variables and quantitative MRCP metrics

For the overall cohort, positive correlations were detected between ROI-based mean cT1, whole liver mean cT1, whole liver median cT1, and liver stiffness and right hepatic duct diameters (*r* = 0.30–0.35; *p* values 0.008–0.02). Whole liver mean cT1, whole liver cT1 IQR, and liver stiffness positively correlated with left hepatic duct diameters (*r* = 0.27–0.38; *p* values 0.004–0.04). There were no other significant correlations between cT1 or liver stiffness measurements and quantitative MRCP metrics.

In the subgroup analysis for patients with AIH, there were positive correlations between all five primary outcome variables and right hepatic bile duct diameters (*r* = 0.45–0.58; *p* values 0.0009–0.01). There also were positive correlations between all five primary outcome variables and left hepatic bile duct diameters (*r* = 0.41–0.69; *p* values < 0.0001–0.02). Whole liver cT1 IQR positively correlated with biliary tree volume, total number and length of strictures, and total number of dilations (*r* = 0.37–0.46; *p* values 0.01–0.04). For the PSC/ASC cohort, none of our primary outcome variables correlated with quantitative 3D MRCP measurements (Supplementary Table 3).

### Associations between outcome variables and clinical metrics

**Fig. 3:**
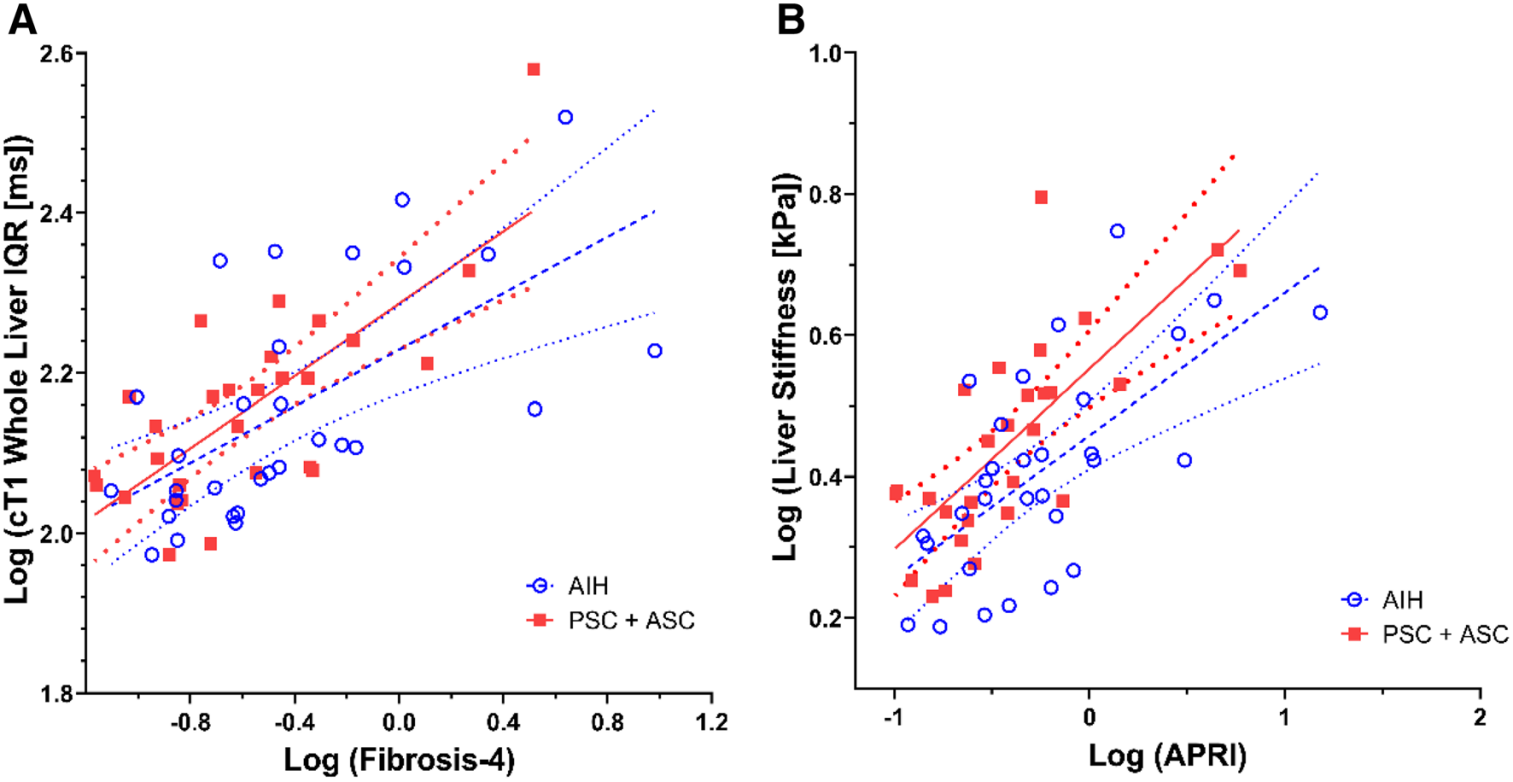
Relationship between outcome variables and two predictor variables. Linear regression lines with 95% confidence intervals shown in blue for AIH group and red for PSC/ASC group. **a** Relationship between log transformed fibrosis-4 score and whole liver cT1 IQR shown. Correlation coefficient for entire cohort was 0.66 (*p* < 0.0001). Correlation coefficient for the AIH group was 0.63 (*p* = 0.0002), while correlation coefficient for the PSC/ASC group was 0.75 (*p* < 0.0001). **b** Relationship between log transformed APRI score and MRE liver stiffness. Correlation coefficient for entire cohort was 0.65 (*p* < 0.0001). Correlation coefficient for the AIH group was 0.65 (*p* = 0.0001), while correlation coefficient for the PSC/ASC group was 0.74 (*p* < 0.0001). *cT1* iron (T2*)-corrected T1, *IQR* interquartile range, *APRI* AST to platelet ratio index, *AIH* autoimmune hepatitis, *PSC* primary sclerosing cholangitis, *ASC* autoimmune sclerosing cholangitis

For the overall cohort, whole liver mean cT1 (*r* = 0.33; *p* = 0.01) and cT1 IQR (*r* = 0.35; *p* = 0.008) positively correlated with age. There were multiple significant associations between cT1 or liver stiffness measurements and laboratory values. Liver stiffness negatively correlated with platelet count (*r* = − 0.34; *p* = 0.01) and positively correlated with all other laboratory values (*r* = 0.33–0.65; *p* values < 0.0001–0.02). Whole liver cT1 IQR was associated with all laboratory variables except alkaline phosphatase and MMP7 (*r* = 0.26–0.66; *p* values < 0.0001–0.04); whole liver cT1 IQR negatively correlated with platelet count (*r* = − 0.50; *p* < 0.0001). ROI-based mean cT1, whole liver mean cT1, and whole liver median cT1 were associated with APRI and FIB4 scores (*r* = 0.27–0.44; *p* values 0.0005–0.04). Figure 3 displays the relationships between log transformed FIB4 score and cT1 IQR and log transformed APRI score and liver stiffness.

For the AIH subgroup, ROI mean cT1, and whole liver mean and median cT1 positively correlated with total bilirubin, APRI, and FIB4 scores (*r* = 0.39–0.64, *p* values 0.0001–0.04) and with AST and MMP7 (*r* = 0.39–0.81, *p* values 0.001–0.03). Whole liver cT1 IQR positively correlated with age (*r* = 0.48, *p* = 0.007). For the PSC/ASC subgroup, whole liver cT1 IQR and liver stiffness positively correlated with ALT, AST, APRI, and FIB4 scores (*r* = 0.46–0.80, *p* values < 0.0001–0.01). Whole liver cT1 IQR correlated with total bilirubin and platelet count (*r* = 0.40 and − 0.53, *p* = 0.04 and 0.004, respectively). Liver stiffness also positively correlated with GGT (*r* = 0.69, *p* < 0.0001).

### Multivariable linear regression

After statistical adjustments for patient age, sex, presence of IBD, specific diagnosis (AIH vs. PSC/ASC), and time from diagnosis to MRI examination, only left hepatic bile duct maximum diameter was statistically significantly associated with whole liver mean cT1 (*p* = 0.01). Eight predictor variables, including seven laboratory values and left hepatic bile duct maximum diameter, were significantly associated with whole liver cT1 IQR (*p* < 0.0001–0.04). Eleven predictor variables, including eight laboratory values and three quantitative MRCP metrics, were significantly associated with liver stiffness (*p* < 0.0001–0.04) (Table 4).

**Table 4:** Multivariable linear regression results across the entire study cohort, associating whole liver mean cT1, whole liver cT1 IQR, and MRE liver stiffness with each independent predictor variable^a^

Predictor variables	Unit of increase examined	Whole liver mean cT1		Whole liver cT1 IQR^c^		MRE liver stiffness^c^	
		β-estimates (ms) [LCL-UCL]	R^2^	Change in Whole liver cT1 IQR^c^	R^2^	Change in MRE liver stiffness^c^	R^2^
Left hepatic bile duct maximum diameter^b^	1 unit (mm)	17.8 [4.80–30.9] (0.01)*	0.44	9% ↑ (0.01)*	0.40	8% ↑ (0.04)*	0.10
Right hepatic bile duct median diameter^b^	1 unit (mm)	6.70 [− 1.90–15.3] (n.s)	0.41	1% ↑ (n.s)	0.26	6% ↑ (0.02)*	0.13
Right hepatic bile duct maximum diameter^b^	1 unit (mm)	5.50 [− 2.20–13.1] (n.s)	0.40	1% ↑ (n.s)	0.26	4% ↑ (0.03)*	0.11
Total bilirubin^c^	10 fold	34.4 [− 24.4–93.2] (n.s)	0.40	50% ↑ (0.003)*	0.38	50% ↑ (0.01)*	0.16
ALT^c^	10 fold	15.5 [− 34.8–65.7] (n.s)	0.39	20% ↑ (n.s)	0.30	80% ↑ (< 0.0001)*	0.37
AST^c^	10 fold	34.3 [− 24.7–93.4] (n.s)	0.40	40% ↑ (0.01)*	0.35	90% ↑ (< 0.0001)*	0.33
GGT^c^	10 fold	6.96 [− 27.2–41.2] (n.s)	0.38	20% ↑ (0.04)*	0.31	50% ↑ (< 0.0001)*	0.43
APRI^c^	10 fold	37.4 [− 3.0–77.7] (n.s)	0.42	40% ↑ (< 0.0001)*	0.50	70% ↑ (< 0.0001)*	0.50
FIB4^c^	10 fold	39.1 [− 1.50–79.8] (n.s)	0.43	50% ↑ (< 0.0001)*	0.50	80% ↑ (< 0.0001)*	0.50
ALP^c^	10 fold	19 [− 49.4–87.5] (n.s)	0.39	60% ↑ (0.001)*	0.40	90% ↑ (0.0003)*	0.26
Platelets^b^	1 unit (x10^9^/L)	− 0.05 [− 0.20–0.10] (n.s)	0.39	9% ↓ (0.0002)*	0.44	9% ↓ (0.002)*	0.20

*p* values presented in parenthesis. R-squared (*R*^2^) values also presented for each regression result. Non-significant predictors of all three outcome variables not presented

*LCL* lower control limit, *UCL* upper control limit, *cT1* iron (T2*)-corrected T1 mapping, *IQR* interquartile range, *MRE* MR elastography, *n.s* not significant (*p* ≥ 0.05), *R*^*2*^ coefficient of determination, *ALT* alanine aminotransferase, *AST* aspartate aminotransferase, *GGT* gamma-glutamyl transferase, *APRI* AST to platelet ratio index, *FIB4* fibrosis-4 score, *ALP* alkaline phosphatase, *PSC* primary sclerosing cholangitis, *ASC* autoimmune sclerosing cholangitis, *AIH* autoimmune hepatitis

*Statistically significant (bold)

^a^Each model adjusted for age, sex, presence of inflammatory bowel disease, specific diagnosis (AIH vs. PSC/ASC), and time from diagnosis to research MRI examination

^b^Variables examined as unit increase

^c^Variables examined as log10 value

Results from stratified [by diagnosis (AIH and PSC/ASC)] linear regression analyses are presented in Supplementary Table 4. Within the AIH group, four quantitative MRCP metrics (*p* values 0.0003–0.001) and five laboratory values (*p* values 0.0001–0.02) were associated with whole liver mean cT1. Five quantitative MRCP metrics (*p* values < 0.0001–0.04) and five laboratory values (*p* values 0.0002–0.02) as well as age (*p* = 0.01) were significantly associated with whole liver cT1 IQR. Four quantitative MRCP metrics (*p* = 0.0003–0.02) and seven laboratory values (*p* < 0.0001–0.047) were significantly associated with MRE liver stiffness. For the PSC/ASC group, no predictor variables were significantly associated with whole liver mean cT1. Five laboratory values (*p* < 0.0001–0.01) were significantly associated with whole liver cT1 IQR, while six laboratory values (*p* < 0.0001–0.001) were significantly associated with MRE liver stiffness. No MRCP metrics were significantly associated with cT1 IQR or MRE liver stiffness.

## Discussion

In a cohort of children and young adults with AILD, we have shown multiple significant associations between quantitative MRI markers (cT1, cT1 IQR, liver stiffness and quantitative MRCP) and between these markers and clinical variables. This suggests that each of the MRI markers and clinical markers under study are, to some degree, responsive to changes of inflammation and/or fibrosis in patients with AILD. Further, it is worth noting that there was no significant correlation between measures of cT1 or liver stiffness and time from AILD diagnosis to MRI examination. This suggests that changes in cT1 measurements and liver stiffness over time are not uniform among AILD patients, and instead may be individually specific.

Quantitative MRCP metrics, inclusive of duct diameters and narrowings, would be expected to be reflective of biliary involvement by disease. Our results support this, with significant differences in quantitative MRCP metrics between the AIH and PSC/ASC subgroup. However, for the overall AILD cohort, only right and left hepatic duct diameters, and none of the other metrics, were significantly correlated with cT1 and liver stiffness, measures that may be more indicative of parenchymal disease. These results contradict prior literature demonstrating associations between liver stiffness and biliary abnormalities measured on MRCP by human observers in the setting of PSC. Tafur et al. [8] demonstrated a positive correlation between liver stiffness measurements and intrahepatic bile duct stricture severity in adult patients with PSC. Bookwalter et al. [9] found a positive correlation between liver stiffness and the presence of a common bile duct stricture and the number of segmental duct strictures in the biliary tree, also in adult patients with PSC. Interestingly, our study failed to demonstrate any significant associations between liver stiffness and quantitative MRCP metrics related to numbers and lengths of strictures or biliary tree volume (a putative measure of upstream duct dilation).

Interestingly, in the AIH subgroup, univariate and multivariable analyses demonstrated multiple quantitative MRCP metrics (e.g., biliary tree volume, number of strictures, and length of strictures) to be significantly associated with cT1 measurements (including cT1 IQR) as well as liver stiffness. These results could reflect mild irregularities in biliary tree structure due to periductal fibrosis in patients with AIH, as reported previously in an adult cohort by Lewin et al. [26]. The absence of such associations on the univariate and multivariable levels in PSC/ASC patients may indicate that large duct injury is not a key driver of fibrosis in PSC/ASC or that large duct changes occur early in the disease before significant changes in cT1 or stiffness occur.

In the overall AILD cohort, measures of cT1, cT1 IQR, and liver stiffness all showed positive associations with numerous laboratory markers of liver disease as well as clinical predictive scores of liver fibrosis (APRI, FIB4). Relationships with these predictive scores were of relatively higher strength for cT1 IQR and liver stiffness than with absolute cT1 measurements. This was similarly the case in the PSC/ASC subgroup where correlations between cT1 IQR and MRE liver stiffness and clinical predictive scores of liver fibrosis were *r* > 0.7. These associations between clinical predictive scores of fibrosis and liver stiffness are not unexpected given prior studies in adults with PSC [27, 28]. However, our findings identify a similar relationship with cT1 IQR, a measure of liver T1 relaxation heterogeneity, suggesting this may be an additional biomarker of liver fibrosis. This potential is further supported by our multivariable analyses which showed cT1 IQR to be less associated with circulating biomarkers of liver inflammation (e.g., AST and ALT) and cholestasis (e.g., GGT and ALP) than liver stiffness is, suggesting cT1 IQR may be less confounded by inflammation and cholestasis as a marker of liver fibrosis.

Our results add to the limited literature evaluating associations between liver T1 relaxation measurements (including cT1) and other markers of chronic liver disease. Previously, Banerjee et al. [6] found a strong positive association between cT1 and increasing fibrosis stage. Hoffman et al. [29] using conventional T1 mapping without T2* correction demonstrated an area under the ROC curve for differentiating early (F0-F2) from advanced (F3-F4) fibrosis of 0.67 for reader 1 and 0.64 for reader 2. Alongside cT1, cT1 IQR has previously been shown to diagnose histologically confirmed disease and decrease following standard of care treatment in pediatric AIH patients [15]. Interestingly, our study suggests that cT1 IQR, as opposed to cT1, may be a better performing biomarker of AILD, although further investigations are needed.

Our study has limitations. First, it is cross-sectional in design, including 58 subjects with AILD. As a result, small but significant correlations could have gone undetected. Such small correlations are unlikely to be clinically useful, however. Second, 25 of the 73 (~ 34%) available baseline MRI examinations could not undergo quantitative MRCP post-processing due to image quality issues, most often motion artifacts. Utilization of faster MRCP methods, such as 3D FSE sequences with compressed sensing, that shorten the acquisition time may reduce the number of datasets that are rejected for quantitative MRCP post-processing. Third, the cT1 metrics were not corrected for fat. The MOLLI sequence used is sensitive to fat as well as to the other features of liver disease such as inflammation and fibrosis. However, cT1 is not routinely corrected for fat because in patients with low liver fat, the signal is dominated by the free-water signal that is generated by the biological processes of inflammation and fibrosis. Thus, there is no need for such fat correction in order to interpret the change in the cT1 signal in response to biology in these populations [30]. Finally, quantitative MRCP metrics and laboratory values were correlated with imaging markers of chronic liver disease and histopathological correlations were not evaluated. Correlating these predictor variables as well as our primary outcomes (particularly liver stiffness and cT1 IQR) with meaningful clinical outcomes would further help establish the value of these various imaging techniques.

In conclusion, we have demonstrated significant associations among automated quantitative MRCP metrics, MRE-derived liver stiffness, T1 relaxation (cT1) measurements, and liver-related laboratory measurements in children and young adults with AILD. Quantitative MRCP metrics related to the diameters of the right and left hepatic bile ducts were significantly associated with measures of cT1 and liver stiffness, especially in AIH patients. Both cT1 IQR and liver stiffness are highly associated with circulating laboratory biomarkers of liver disease, and in particular clinical fibrosis scores. Our results also suggest that T1 relaxation heterogeneity (cT1 IQR) may be a novel marker of liver fibrosis which may be less impacted by cholestasis and inflammation than MR elastography.

## Supplementary Information

Below is the link to the electronic supplementary material.

Supplementary file1 (DOCX 32 KB)
