# Correction to: Associations between MRI T1 mapping, liver stiffness, quantitative MRCP, and laboratory biomarkers in children and young adults with autoimmune liver disease

**DOI:** 10.1007/s00261-022-03437-0

**Published:** 2022-02-16

**Authors:** Neeraja Mahalingam, Andrew T. Trout, Deep B. Gandhi, Rashmi D. Sahay, Ruchi Singh, Alexander G. Miethke, Jonathan R. Dillman

**Affiliations:** 1 Department of Radiology, Imaging Research Center, Cincinnati Children’s Hospital Medical Center, 250 Albert Sabin Way, Cincinnati, OH USA; 2 Department of Radiology, Cincinnati Children’s Hospital Medical Center, Cincinnati, OH USA; 3 Department of Radiology, University of Cincinnati College of Medicine, Cincinnati, OH USA; 4 Department of Pediatrics, University of Cincinnati College of Medicine, Cincinnati, OH USA; 5 Division of Biostatistics and Epidemiology, Cincinnati Children’s Hospital Medical Center, Cincinnati, OH USA; 6 Center for Autoimmune Liver Disease (CALD), Cincinnati Children’s Hospital Medical Center, Cincinnati, OH USA; 7 Division of Gastroenterology, Hepatology, and Nutrition, Cincinnati Children’s Hospital Medical Center, Cincinnati, OH USA

## Correction to: Abdominal Radiology (2022) 47:672–683 10.1007/s00261-021-03378-0

The article “Associations between MRI T1 mapping, liver stiffness, quantitative MRCP, and laboratory biomarkers in children and young adults with autoimmune liver disease”, written Neeraja Mahalingam, Andrew T. Trout, Deep B. Gandhi, Rashmi D. Sahay, Ruchi Singh, Alexander G. Miethke, Jonathan R. Dillman, was originally published electronically on the publisher's internet portal on 21 December 2021 without open access. With the author(s)' decision to opt for Open Choice the copyright of the article changed on 19 January 2022 to © The Author(s) 2021 and the article is forthwith distributed under a Creative Commons Attribution 4.0 International License, which permits use, sharing, adaptation, distribution and reproduction in any medium or format, as long as you give appropriate credit to the original author(s) and the source, provide a link to the Creative Commons licence, and indicate if changes were made. The images or other third party material in this article are included in the article's Creative Commons licence, unless indicated otherwise in a credit line to the material. If material is not included in the article's Creative Commons licence and your intended use is not permitted by statutory regulation or exceeds the permitted use, you will need to obtain permission directly from the copyright holder. To view a copy of this licence, visit http://creativecommons.org/licenses/ by/4.0.

The original article has been corrected.

