# Supplementary material for: Associations between MRI T1 mapping, liver stiffness, quantitative MRCP, and laboratory biomarkers in children and young adults with autoimmune liver disease
Source: Abdom Radiol (NY). 2021 Dec 21;47(2):672–83. doi: 10.1007/s00261-021-03378-0 (PMC8847161; doi:10.1007/s00261-021-03378-0)
Supplement: Supplementary file 1 — Supplementary file1 (DOCX 32 KB) [file 261_2021_3378_MOESM1_ESM.docx]

**Supplementary Material**

**Supplementary Table 1:** Log transformed variables.

| **MRE** | MRE liver stiffness |
| --- | --- |
| **cT1** | Whole liver cT1 IQR |
| **MRCP+ metrics** | Biliary tree volume |
|  | Total number of ducts |
|  | Total length of ducts |
|  | Total number of strictures |
|  | Total length of strictures |
|  | Total number dilations |
|  | Total length of dilations |
| **Laboratory results** | Total bilirubin |
|  | ALT |
|  | AST |
|  | GGT |
|  | APRI |
|  | FIB4 |
|  | ALP |
|  | MMP7 |

MRE: MR elastography (kPa); cT1: iron (T2*)-corrected T1 mapping (ms); IQR: interquartile range; ALT: alanine aminotransferase (units/L); AST: aspartate aminotransferase (units/L); GGT: gamma-glutamyl transferase (units/L); APRI: AST to platelet ratio index; FIB4: fibrosis-4 score; ALP: alkaline phosphatase (units/L); MMP7: matrix metalloproteinase 7 (units/L)

**Supplemental Table 2:** Univariate correlations between liver stiffness and cT1 measurements and predictor variables in autoimmune hepatitis (AIH) group (n=30). Pearson correlation coefficients (r) are presented with 95% confidence intervals in brackets and p-values in parentheses.

| **Predictor Variables** | **ROI-based Mean cT1** | **Whole Liver Mean cT1** | **Whole Liver Median cT1** | **Whole Liver cT1 IQR**^§^ | **MRE Liver Stiffness**^§^ |
| --- | --- | --- | --- | --- | --- |
| CBD median diameter | 0.11  [-0.26, 0.45]  (n.s) | 0.21  [-0.16, 0.53]  (n.s) | 0.21  [-0.16, 0.53]  (n.s) | 0.36  [0, 0.63]  (n.s) | 0.30  [-0.06, 0.60]  (n.s) |
| CBD maximum diameter | 0.24  [-0.13, 0.55]  (n.s) | 0.30  [-0.07, 0.59]  (n.s) | 0.32  [-0.04, 0.61]  (n.s) | 0.26  [-0.11, 0.57]  (n.s) | 0.27  [-0.10, 0.57]  (n.s) |
| Left hepatic bile duct median diameter | **0.43**  **[0.09, 0.69]**  **(0.02)^*^** | **0.62**  **[0.33, 0.80]**  **(0.0003)^*^** | **0.57**  **[0.26, 0.77]**  **(0.001)^*^** | **0.55**  **[0.24, 0.76]**  **(0.002)^*^** | **0.41**  **[0.06, 0.67]**  **(0.02)^*^** |
| Left hepatic bile duct maximum diameter | **0.41**  **[0.06, 0.67]**  **(0.02)^*^** | **0.62**  **[0.33, 0.80]**  **(0.0003)^*^** | **0.53**  **[0.21, 0.75]**  **(0.003)^*^** | **0.69**  **[0.44, 0.84]**  **(<0.0001)^*^** | **0.62**  **[0.33, 0.80]**  **(0.0003)^*^** |
| Right hepatic bile duct median diameter | **0.49**  **[0.16, 0.73]**  **(0.006)^*^** | **0.58**  **[0.27, 0.78]**  **(0.0009)^*^** | **0.53**  **[0.21, 0.75]**  **(0.003)^*^** | **0.46**  **[0.11, 0.70]**  **(0.01)^*^** | **0.54**  **[0.22, 0.75]**  **(0.002)^*^** |
| Right hepatic bile duct maximum diameter | **0.48**  **[0.14, 0.72]**  **(0.008)^*^** | **0.57**  **[0.27, 0.77]**  **(0.0009)^*^** | **0.51**  **[0.19, 0.74]**  **(0.004)^*^** | **0.45**  **[0.11, 0.70]**  **(0.01)^*^** | **0.48**  **[0.15, 0.72]**  **(0.007)^*^** |
| Biliary tree volume^§^ | 0.01  [-0.35, 0.37]  (n.s) | 0.15  [-0.23, 0.48]  (n.s) | 0.05  [-0.32, 0.40]  (n.s) | **0.38**  **[0.03, 0.65]**  **(0.04)^*^** | 0.21  [-0.16, 0.53]  (n.s) |
| Total number of ducts^§^ | -0.13  [-0.46, 0.25]  (n.s) | -0.04  [-0.40, 0.32]  (n.s) | -0.10  [-0.45, 0.27]  (n.s) | 0.15  [-0.22, 0.49]  (n.s) | 0.002  [-0.36, 0.36]  (n.s) |
| Total length of ducts^§^ | -0.07  [-0.42, 0.30]  (n.s) | 0.03  [-0.34, 0.38]  (n.s) | -0.04  [-0.40, 0.32]  (n.s) | 0.25  [-0.12, 0.56]  (n.s) | 0.08  [-0.28, 0.43]  (n.s) |
| Total number of strictures^§^ | 0.14  [-0.24, 0.47]  (n.s) | 0.32  [-0.05, 0.61]  (n.s) | 0.20  [-0.18, 0.52]  (n.s) | **0.46**  **[0.12, 0.70]**  **(0.01)^*^** | 0.29  [-0.08, 0.59]  (n.s) |
| Total length of strictures^§^ | 0.12  [-0.25, 0.46]  (n.s) | 0.32  [-0.05, 0.61]  (n.s) | 0.19  [-0.18, 0.52]  (n.s) | **0.45**  **[0.11, 0.70]**  **(0.01)^*^** | 0.28  [-0.09, 0.58]  (n.s) |
| Total number of dilations^§^ | 0.09  [-0.27, 0.44]  (n.s) | 0.21  [-0.17, 0.53]  (n.s) | 0.12  [-0.25, 0.46]  (n.s) | **0.37**  **[0.01, 0.64]**  **(0.04)^*^** | 0.32  [-0.05, 0.61]  (n.s) |
| Total length of dilations^§^ | 0.11  [-0.26, 0.46]  (n.s) | 0.23  [-0.14, 0.54]  (n.s) | 0.13  [-0.24, 0.47]  (n.s) | 0.31  [-0.05, 0.60]  (n.s) | 0.29  [-0.08, 0.59]  (n.s) |
| Age | 0.12  [-0.25, 0.46]  (n.s) | 0.31  [-0.06, 0.60]  (n.s) | 0.14  [-0.23, 0.48]  (n.s) | **0.48**  **[0.15, 0.72]**  **(0.007)^*^** | 0.11  [-0.26, 0.45]  (n.s) |
| Time from diagnosis to MRI | -0.21  [-0.55, 0.19]  (n.s) | -0.17  [-0.52, 0.22]  (n.s) | -0.24  [-0.57, 0.15]  (n.s) | -0.04  [-0.41, 0.35]  (n.s) | -0.15  [-0.50, 0.25]  (n.s) |
| Total bilirubin^§^ | **0.39**  **[0.03, 0.66]**  **(0.04)^*^** | **0.59**  **[0.29, 0.79]**  **(0.0006)^*^** | **0.41**  **[0.06, 0.67]**  **(0.02)^*^** | **0.64**  **[0.36, 0.81]**  **(0.0002)^*^** | **0.37**  **[0.01, 0.64]**  **(0.04)^*^** |
| ALT^§^ | 0.30  [-0.07, 0.60]  (n.s) | 0.30  [-0.07, 0.59]  (n.s) | 0.31  [-0.06, 0.60]  (n.s) | 0.20  [-0.17, 0.53]  (n.s) | 0.36  [0, 0.64]  (n.s) |
| AST^§^ | **0.39**  **[0.03, 0.66]**  **(0.03)^*^** | **0.42**  **[0.07, 0.68]**  **(0.02)^*^** | **0.45**  **[0.10, 0.70]**  **(0.01)^*^** | 0.26  [-0.12, 0.56]  (n.s) | **0.38**  **[0.02, 0.65]**  **(0.04)^*^** |
| GGT^§^ | 0.23  [-0.15, 0.54]  (n.s) | 0.25  [-0.13, 0.56]  (n.s) | 0.23  [-0.14, 0.55]  (n.s) | **0.42**  **[0.07, 0.68]**  **(0.02)^*^** | **0.45**  **[0.11, 0.70]**  **(0.01)^*^** |
| Platelets | -0.33  [-0.62, 0.03]  (n.s) | **-0.50**  **[-0.73, -0.18]**  **(0.004)^*^** | **-0.40**  **[-0.66, -0.05]**  **(0.03)^*^** | **-0.58**  **[-0.78, -0.27]**  **(0.0009)^*^** | **-0.58**  **[-0.78, -0.28]**  **(0.0008)^*^** |
| APRI^§^ | **0.48**  **[0.15, 0.72]**  **(0.007)^*^** | **0.62**  **[0.34, 0.80]**  **(0.0002)^*^** | **0.58**  **[0.28, 0.78]**  **(0.0008)^*^** | **0.55**  **[0.24, 0.76]**  **(0.002)^*^** | **0.65**  **[0.38, 0.82]**  **(0.0001)^*^** |
| FIB4^§^ | **0.47**  **[0.14, 0.71]**  **(0.008)^*^** | **0.64**  **[0.37, 0.81]**  **(0.0001)^*^** | **0.56**  **[0.25, 0.77]**  **(0.001)^*^** | **0.63**  **[0.34, 0.80]**  **(0.0002)^*^** | **0.62**  **[0.34, 0.80]**  **(0.0002)^*^** |
| ALP^§^ | -0.06  [-0.41, 0.31]  (n.s) | -0.11  [-0.45, 0.26]  (n.s) | -0.03  [-0.39, 0.33]  (n.s) | -0.02  [-0.38, 0.34]  (n.s) | 0.19  [-0.18, 0.52]  (n.s) |
| MMP7^§^ | **0.74**  **[0.28, 0.92]**  **(0.006)^*^** | **0.81**  **[0.44, 0.94]**  **(0.001)^*^** | **0.73**  **[0.28, 0.92]**  **(0.007)^*^** | 0.50  [-0.10, 0.84]  (n.s) | 0.49  [-0.11, 0.83]  (n.s) |

cT1: iron (T2*)-corrected T1 mapping; ROI: region of interest; IQR: interquartile range; MRE: MR elastography; n.s: not significant (p≥0.05); CBD: common bile duct; ALT: alanine aminotransferase (units/L); AST: aspartate aminotransferase (units/L); GGT: gamma-glutamyl transferase (units/L); APRI: AST to platelet ratio index; FIB4: fibrosis-4 score; ALP: alkaline phosphatase (units/L); MMP7: matrix metalloproteinase 7 (units/L)

^*^statistically significant (bold)

^§^variables examined as log10 value

**Supplemental Table 3:** Univariate correlations between liver stiffness and cT1 measurements and predictor variables in the primary sclerosing cholangitis (PSC) / autoimmune sclerosing cholangitis (ASC) group (n=28). Pearson correlation coefficients (r) are presented with 95% confidence intervals in brackets and p-values in parentheses.

| **Predictor Variables** | **ROI-based Mean cT1** | **Whole Liver Mean cT1** | **Whole Liver Median cT1** | **Whole Liver cT1 IQR**^§^ | **MRE Liver Stiffness**^§^ |
| --- | --- | --- | --- | --- | --- |
| CBD median diameter | 0.04  [-0.34, 0.41]  (n.s) | 0.10  [-0.29, 0.45]  (n.s) | 0.01  [-0.37, 0.38]  (n.s) | 0.15  [-0.23, 0.50]  (n.s) | -0.30  [-0.60, 0.09]  (n.s) |
| CBD maximum diameter | 0.09  [-0.29, 0.45]  (n.s) | 0.13  [-0.25, 0.48]  (n.s) | 0.04  [-0.34, 0.41]  (n.s) | 0.22  [-0.17, 0.54]  (n.s) | -0.07  [-0.43, 0.31]  (n.s) |
| Left hepatic bile duct median diameter | -0.03  [-0.42, 0.37]  (n.s) | 0.07  [-0.34, 0.45]  (n.s) | -0.03  [-0.42, 0.37]  (n.s) | 0.10  [-0.31, 0.47]  (n.s) | 0.08  [-0.33, 0.46]  (n.s) |
| Left hepatic bile duct maximum diameter | 0.04  [-0.36, 0.43]  (n.s) | 0.16  [-0.25, 0.52]  (n.s) | 0.06  [-0.35, 0.44]  (n.s) | 0.04  [-0.36, 0.43]  (n.s) | -0.14  [-0.51, 0.27]  (n.s) |
| Right hepatic bile duct median diameter | 0.17  [-0.23, 0.51]  (n.s) | 0.08  [-0.31, 0.45]  (n.s) | 0.06  [-0.33, 0.43]  (n.s) | -0.04  [-0.41, 0.35]  (n.s) | 0.14  [-0.26, 0.49]  (n.s) |
| Right hepatic bile duct maximum diameter | 0.19  [-0.21, 0.53]  (n.s) | 0.10  [-0.29, 0.46]  (n.s) | 0.08  [-0.31, 0.45]  (n.s) | -0.02  [-0.40, 0.36]  (n.s) | 0.15  [-0.24, 0.50]  (n.s) |
| Biliary tree volume^§^ | 0.05  [-0.33, 0.42]  (n.s) | 0.13  [-0.26, 0.48]  (n.s) | 0.05  [-0.33, 0.41]  (n.s) | -0.12  [-0.47, 0.27]  (n.s) | -0.12  [-0.48, 0.26]  (n.s) |
| Total number of ducts^§^ | 0.12  [-0.26, 0.48]  (n.s) | 0.19  [-0.20, 0.53]  (n.s) | 0.14  [-0.24, 0.49]  (n.s) | -0.17  [-0.51, 0.22]  (n.s) | -0.13  [-0.48, 0.26]  (n.s) |
| Total length of ducts^§^ | 0.14  [-0.24, 0.49]  (n.s) | 0.20  [-0.18, 0.54]  (n.s) | 0.15  [-0.24, 0.49]  (n.s) | -0.17  [-0.51, 0.22]  (n.s) | -0.16  [-0.50, 0.22]  (n.s) |
| Total number of strictures^§^ | -0.07  [-0.43, 0.31]  (n.s) | 0.01  [-0.37, 0.38]  (n.s) | -0.07  [-0.43, 0.31]  (n.s) | -0.13  [-0.48, 0.25]  (n.s) | -0.06  [-0.42, 0.32]  (n.s) |
| Total length of strictures^§^ | -0.03  [-0.40, 0.35]  (n.s) | 0.04  [-0.34, 0.41]  (n.s) | -0.02  [-0.39, 0.35]  (n.s) | -0.14  [-0.49, 0.24]  (n.s) | -0.16  [-0.50, 0.23]  (n.s) |
| Total number of dilations^§^ | 0.04  [-0.34, 0.40]  (n.s) | 0.12  [-0.27, 0.47]  (n.s) | 0.07  [-0.31, 0.43]  (n.s) | -0.09  [-0.45, 0.29]  (n.s) | -0.008  [-0.38, 0.37]  (n.s) |
| Total length of dilations^§^ | 0.04  [-0.34, 0.41]  (n.s) | 0.14  [-0.24, 0.49]  (n.s) | 0.06  [-0.32, 0.42]  (n.s) | -0.08  [-0.44, 0.31]  (n.s) | -0.05  [-0.42, 0.33]  (n.s) |
| Age | 0.28  [-0.10, 0.59]  (n.s) | 0.35  [-0.02, 0.64]  (n.s) | 0.27  [-0.12, 0.58]  (n.s) | 0.13  [-0.25, 0.48]  (n.s) | -0.25  [-0.57, 0.14]  (n.s) |
| Time from diagnosis to MRI | -0.11  [-0.46, 0.28]  (n.s) | -0.16  [-0.50, 0.23]  (n.s) | -0.19  [-0.52, 0.20]  (n.s) | 0.23  [-0.15, 0.56]  (n.s) | 0.19  [-0.20, 0.52]  (n.s) |
| Total bilirubin^§^ | -0.10  [-0.45, 0.29]  (n.s) | -0.04  [-0.40, 0.34]  (n.s) | -0.09  [-0.45, 0.29]  (n.s) | **0.40**  **[0.03, 0.67]**  **(0.04)^*^** | 0.31  [-0.07, 0.61]  (n.s) |
| ALT^§^ | 0.12  [-0.26, 0.47]  (n.s) | 0.12  [-0.26, 0.48]  (n.s) | 0.08  [-0.30, 0.44]  (n.s) | **0.46**  **[0.11, 0.71]**  **(0.01)^*^** | **0.80**  **[0.60, 0.90]**  **(<0.0001)^*^** |
| AST^§^ | -0.004  [-0.38, 0.37]  (n.s) | 0.004  [-0.34, 0.41]  (n.s) | -0.02  [-0.39, 0.35]  (n.s) | **0.59**  **[0.27, 0.79]**  **(0.001)^*^** | **0.78**  **[0.58, 0.90]**  **(<0.0001)^*^** |
| GGT^§^ | 0.11  [-0.27, 0.47]  (n.s) | -0.01  [-0.39, 0.36]  (n.s) | 0.03  [-0.34, 0.40]  (n.s) | 0.17  [-0.22, 0.51]  (0.39) | **0.69**  **[0.43, 0.85]**  **(<0.0001)^*^** |
| Platelets | 0.22  [-0.16, 0.55]  (n.s) | 0.12  [-0.27, 0.47]  (n.s) | 0.22  [-0.16, 0.55]  (n.s) | **-0.53**  **[-0.75, -0.19]**  **(0.004)^*^** | -0.29  [-0.60, 0.09]  (n.s) |
| APRI^§^ | -0.05  [-0.41, 0.33]  (n.s) | -0.005  [-0.38, 0.37]  (n.s) | -0.09  [-0.45, 0.29]  (n.s) | **0.72**  **[0.47, 0.86]**  **(<0.0001)^*^** | **0.74**  **[0.51, 0.87]**  **(<0.0001)^*^** |
| FIB4^§^ | 0.01  [-0.37, 0.38]  (n.s) | 0.07  [-0.31, 0.43]  (n.s) | -0.03  [-0.40, 0.35]  (n.s) | **0.75**  **[0.52, 0.87]**  **(<0.0001)^*^** | **0.71**  **[0.46, 0.86]**  **(<0.0001)^*^** |
| ALP^§^ | -0.18  [-0.52, 0.21]  (n.s) | -0.26  [-0.57, 0.13]  (n.s) | -0.24  [-0.57, 0.14]  (n.s) | 0.32  [-0.06, 0.62]  (n.s) | **0.58**  **[0.26, 0.78]**  **(0.001)^*^** |
| MMP7^§^ | 0.01  [-0.66, 0.67]  (n.s) | -0.05  [-0.69, 0.63]  (n.s) | 0.10  [-0.61, 0.72]  (n.s) | 0.02  [-0.65, 0.67]  (n.s) | 0.62  [-0.07, 0.91]  (n.s) |

cT1: iron (T2*)-corrected T1 mapping; ROI: region of interest; IQR: interquartile range; MRE: MR elastography; n.s: not significant (p≥0.05); CBD: common bile duct; ALT: alanine aminotransferase (units/L); AST: aspartate aminotransferase (units/L); GGT: gamma-glutamyl transferase (units/L); APRI: AST to platelet ratio index; FIB4: fibrosis-4 score; ALP: alkaline phosphatase (units/L); MMP7: matrix metalloproteinase 7 (units/L)

^*^statistically significant (bold)

^§^variables examined as log10 value

**Supplementary Table 4:** Multivariable linear regression results across the autoimmune hepatitis (AIH) group (n=30) and the primary sclerosing cholangitis (PSC) / autoimmune sclerosing cholangitis (ASC) group (n=28), associating whole liver mean cT1, whole liver cT1 IQR, and MRE liver stiffness with each independent predictor variable^€^. P-values presented in parenthesis. R-squared values also presented for each regression result. Non-significant predictors of all 3 outcome variables not presented.

|  |  | **Whole liver mean cT1** | | | | **Whole liver cT1 IQR**^§^ | | | | **MRE Liver stiffness**^§^ | | | |
| --- | --- | --- | --- | --- | --- | --- | --- | --- | --- | --- | --- | --- | --- |
| **Predictor Variables** | **Unit increase examined** | **AIH** | | **PSC/ASC** | | **AIH** | | **PSC/ASC** | | **AIH** | | **PSC/ASC** | |
|  |  | **β-estimates (ms)**  **[LCL, UCL]** | **R^2^** | **β-estimates (ms)**  **[LCL, UCL]** | **R^2^** | **%Change** | **R^2^** | **%Change** | **R^2^** | **%Change** | **R^2^** | **%Change** | **R^2^** |
| Biliary tree volume^§^ | 10 fold | 39.0 [-62.9, 140]  (n.s) | 0.02 | 22.9 [-50.2, 96.0]  (n.s) | 0.02 | 50% ↑  **(0.04)^*^** | 0.15 | 8% ↓  (n.s) | 0.01 | 30% ↑  (n.s) | 0.04 | 10% ↓  (n.s) | 0.02 |
| Left hepatic bile duct median diameter^¥^ | 1 unit (mm) | 45.4 [22.9, 67.9]  **(0.0003)^*^** | 0.38 | 3.47 [-18.6, 25.5]  (n.s) | 0.001 | 20% ↑  **(0.002)^*^** | 0.31 | 2% ↑  (n.s) | 0.01 | 10% ↑  **(0.02)^*^** | 0.17 | 2% ↑  (n.s) | 0.01 |
| Left hepatic bile duct maximum diameter^¥^ | 1 unit (mm) | 36.4 [18.5, 54.4]  **(0.0003)^*^** | 0.38 | 7.30 [-11.7, 26.3]  (n.s) | 0.03 | 20% ↑  **(<0.0001)^*^** | 0.48 | 0.9% ↑  (n.s) | 0.001 | 20% ↑  **(0.0003)^*^** | 0.38 | 4% ↓  (n.s) | 0.02 |
| Right hepatic bile duct median diameter^¥^ | 1 unit (mm) | 23.1 [10.4, 35.7]  **(0.001)^*^** | 0.33 | 2.46 [-10.2, 15.2]  (n.s) | 0.01 | 8% ↑  **(0.01)^*^** | 0.21 | 0.5% ↓  (n.s) | 0.001 | 9% ↑  **(0.002)^*^** | 0.29 | 2% ↑  (n.s) | 0.02 |
| Right hepatic bile duct maximum diameter^¥^ | 1 unit (mm) | 19.9 [8.92, 30.9]  **(0.001)^*^** | 0.33 | 2.60 [-8.30, 13.5]  (n.s) | 0.01 | 6% ↑  **(0.01)^*^** | 0.20 | 0.2% ↓  (n.s) | 0.001 | 7% ↑  **(0.01)^*^** | 0.23 | 2% ↑  (n.s) | 0.02 |
| Age^¥^ | 1 unit  (year) | 5.63 [-1.11, 12.4]  (n.s) | 0.09 | 7.05 [-0.44, 17.0]  (n.s) | 0.13 | 4% ↑  **(0.01)^*^** | 0.23 | 1% ↑  (n.s) | 0.02 | 0.8% ↑  (n.s) | 0.01 | 3% ↓  (n.s) | 0.06 |
| Total bilirubin^§^ | 10 fold | 138 [65.3, 211]  **(0.001)^*^** | 0.35 | -7.40 [-88.3, 73.5]  (n.s) | 0.001 | 80% ↑  **(0.0002)^*^** | 0.40 | 40% ↑  (n.s) | 0.16 | 40% ↑  **(0.04)*** | 0.14 | 40% ↑  (n.s) | 0.09 |
| ALT^§^ | 10 fold | 63.8 [-15.6, 143]  (n.s) | 0.09 | 23.5 [-51.8, 98.8]  (n.s) | 0.02 | 20% ↑  (n.s) | 0.04 | 40% ↑  **(0.01)^*^** | 0.22 | 40% ↑  **(0.047)*** | 0.13 | 110% ↑  **(<0.0001)^*^** | 0.63 |
| AST^§^ | 10 fold | 111 [18.6, 202]  **(0.02)*** | 0.18 | 9.91 [-87.2, 107]  (n.s) | 0.002 | 30% ↑  (n.s) | 0.07 | 80% ↑  **(0.001)^*^** | 0.34 | 50% ↑  **(0.04)*** | 0.14 | 150% ↑  **(<0.0001)^*^** | 0.61 |
| GGT^§^ | 10 fold | 48.6 [-25.8, 123]  (n.s) | 0.06 | -1.65 [-46.3, 43.1]  (n.s) | 0.002 | 40% ↑  **(0.02)^*^** | 0.18 | 8% ↑  (n.s) | 0.03 | 40% ↑  **(0.01)^*^** | 0.21 | 50% ↑ **(<0.0001)^*^** | 0.48 |
| APRI^§^ | 10 fold | 109 [56.1, 163]  **(0.0002)^*^** | 0.39 | -0.46 [-65.9, 65.0]  (n.s) | 0.01 | 50% ↑  **(0.002)^*^** | 0.31 | 60% ↑  **(<0.0001)^*^** | 0.51 | 60% ↑  **(<0.0001)^*^** | 0.42 | 80% ↑  **(<0.0001)^*^** | 0.55 |
| FIB4^§^ | 10 fold | 105 [57.0, 154]  **(0.0001)^*^** | 0.41 | 12.7 [-55.1, 80.4]  (n.s) | 0.01 | 50% ↑  **(0.0002)^*^** | 0.39 | 70% ↑  **(<0.0001)^*^** | 0.56 | 50% ↑  **(0.0002)^*^** | 0.39 | 80% ↑  **(<0.0001)^*^** | 0.50 |
| ALP^§^ | 10 fold | -38.2 [-170, 93.6]  (n.s) | 0.01 | -55.7 [-140, 29.0]  (n.s) | 0.07 | 3% ↓  (n.s) | 0.001 | 30% ↑  (n.s) | 0.10 | 30% ↑  (n.s) | 0.04 | 80% ↑  **(0.001)^*^** | 0.34 |
| Platelets^¥^ | 1 unit  (x10^9^/L) | -0.46 [-0.76, -0.15]  **(0.004)^*^** | 0.26 | 0.07 [-0.16, 0.29]  (n.s) | 0.01 | 0.2% ↓  **(0.001)^*^** | 0.33 | 0.1% ↓  **(0.004)^*^** | 0.28 | 0.2% ↓  **(0.001)^*^** | 0.34 | 0.01 ↑  (n.s) | 0.08 |

LCL: lower control limit; UCL: upper control limit; cT1: iron (T2*)-corrected T1 mapping; IQR: interquartile range; MRE: MR elastography; n.s: not significant (p≥0.05); R^2^: coefficient of determination; ALT: alanine aminotransferase; AST: aspartate aminotransferase; GGT: gamma-glutamyl transferase; APRI: AST to platelet ratio index; FIB4: fibrosis-4 score; ALP: alkaline phosphatase;

^€^each model adjusted for age, sex, presence of inflammatory bowel disease, and time from diagnosis to research MRI examination.

^¥^variables examined as unit increase;

^§^variables examined as log10 value;

^*^statistically significant (bold)
